# On the importance of metadata when sharing and opening data

**DOI:** 10.1186/s12863-022-01095-1

**Published:** 2022-11-12

**Authors:** Francois Sabot

**Affiliations:** Open Science Mission, French National Research Institute for Sustainable Development – IRD, Occitanie, France

## Abstract

While data sharing increases, most open data are difficult to re-use or to identify due to the lack of related metada. In this editorial, I discussed about the importance of those metadata in the context of genomic, and why they are mandatory to ensure the success of data sharing.

## Introduction

Data in research are by now the *“nerf de la guerre”*, as we say in French, meaning the crux of every analyses and projects, before and after. The OECD defined *“research data” […] as factual records (numerical scores, textual records, images and sounds) used as primary sources for scientific research, and that are commonly accepted in the scientific community as necessary to validate research findings. A research data set constitutes a systematic, partial representation of the subject being investigated”* [1]. Under this quite large definition, we can assume that all the “things” we generate during any study as a result is a data: sequencing products/reads, genome or transcriptome assembly, mapping files, SNP, read counts, annotation files… In genomics in particular, we generate a whole bunch of those data. In recent years, due to the rising tide of the Open Science in genomics and in science in general, we all started to share massively all our data. In this regard, most of us try to respect the FAIR usage (Findable, Accessible, Interoperable, Reusable)[2] However, data are “only” the tip of the iceberg: to release their true potential and allow their full re-usage, they must be associated with their metadata.

What does metadata cover? It means whatever is describing data, without being the data themselves. Metadata point the way to a better understanding of how the data were generated, what level of confidence one can put on them, and additionally allow optimization of their discovery and their reuse (the F and R in FAIR, respectively). Metadata generally consists of of an attribute and of its value, such as “genus: Homo”, generally with a set of fixed attributes (e.g. the Darwin Core [3], or the Minimum Information about a Genome Sequence MIGS from the Genomic Standards Consortium [4]).

Saying that, plenty of things can be metadata, some obvious and some not. Lagoze et al. [5] described seven types of metadata; however, all of them are not really relevant in our genomic research context. Here, I categorize metadata into three types, overlapping with in language from Lagoze et al. [5], and while each of them have specific advantages in various situations, they are all mandatory to ensure a real open data state and a complete FAIR level.

## Type 1: Sampling metadata

This is what Lagoze called the “provenance and identification” metadata, and they are mandatory in the Darwin Core and MIGS. All data indeed have an origin: for instance, sequences came from DNA/RNA sequencing from samples (or population of) in a specific location. This location can be a natural one (or an old agronomical variety, or whatever outside a lab), and thus being described by GPS coordinates (or at least the common geographical name of the region where the samples belong). If it is an non-natural one (i.e. long-lasting lab strains for bacteria, yeasts, nematodes or e.g. *Drosophila melanogaster*), their “genebank” origin knowledge is of importance. In the case of “from *natura*” samples, having the location metadata allows to validate many things: Nagoya protocol respect (i.e. the equal sharing of benefits if any), sampling methodology, coherence of the sampling, post-study availability of samples, and so on. For human tissue sample, the case is more complex: depending on the type of clinical analyses (cancer, cell, genotyping…), the metadata change (e.g. Human Cell Atlas, Cancer Genomics Cloud). In addition, they all request anonymization of the donor, while guaranteeing that we can come back to the patient if needed. Outside of the technical validation, these information allow also other scientists to re-use your data for another analysis: adding samples from other origin for their own analysis, or rethink completely the data for a new study. In this last case, for instance, a dataset created for SNP diversity can be used for inference of demography, based on GPS location data.

## Type 2: Handling metadata

All data came from experimentation. By experimentation, I mean here any modality to obtain the data (not to analyze or transform them, see below), and is recorded as administrative as well as structural metadata by Lagoze. Even biodiversity analysis has experimental procedures: enumerating birds in a given area can be done through different methods (date, hour, pictures, counting methodology) and thus provide different information (or level of). The way you acquired the data implies biases in your data, that can be of different types and orders. For instance, having a genome sequence using a TrueSeqv3 from Illumina has not the same bias as a LSK-109 kit from Oxford Nanopore Technologies (for output, depth bias profile, error rate/type or read length e.g.).

## Type 3: Processing metadata (structural)

Most of the data we work with are transformed during the studies, or even are already transformed data when we used them. The most perfect example is the sequence data: none of the sequencers provides ATGC information. They provide colors (Illumina, Pacific Biosciences, Sanger) or electric signal (ONT) that are processed through different softwares in an ATGC sequence format. While it is not crucial for Illumina or PacBio data, such transformation is critical for ONT, and having the basecaller version and options is mandatory to understand biases and limits of the analysis [6]. While this example seems tightly linked to the previous paragraph, here I would like to insist on the analysis/transformation part and not on the acquisition one. Analysis metadata are of high importance in secondary analyses, especially when a bunch of softwares with many many different options are involved (e.g. in the case of SNP calling, or for genome/transcriptome assembly). Indeed, changing a single parameter or version may modify the whole results and interpretation of data, thus knowing how the initial data were treated to obtain the ones you are working on is of high importance [7, 8]. Thus, the metadata related to options, versions, and post-treatments are as important as the sampling choice for the understanding of data and their interpretation.

## Conclusion

In these times of “Publish or Perish”, the idea of “Share and Flourish” is a nice alternative, and foresees possibilities of growing for research results. Indeed, sharing allows to realize the dream of Bernard de Chartes (XII^th^ century), “being on the shoulders of giants”. It means that you can rely on the whole previous research findings and data for your own research, and help other researchers to perform new, up-to-date research. Sharing genomic data has thus plenty of advantages, and is a win-win situation, even outside of any pure science consideration: such as reproducibility and acknowledgments. However, sharing high-quality data is useless if good metadata are not linked with it. It is more or less like having flatpack furniture without its assembly instructions: you cannot do anything without it, it stays an unusable bunch of planks and dowels. If sharing is caring, then providing good metadata is the biscuit base of the cheesecake.

## Data Availability

Not applicable.

## Abbreviations

OECD: organization for Economic Co-operation and Development
SNP: single-Nucleotide Polymorphism
FAIR: findable, Accessible, Interoperable, Reusable
MIGS: m Information about Genome Sequence
ONT: oxford Nanopore Technologies
